# Continuously improving outcome over time after second allogeneic stem cell transplantation in relapsed acute myeloid leukemia: an EBMT registry analysis of 1540 patients

**DOI:** 10.1038/s41408-024-01060-4

**Published:** 2024-05-02

**Authors:** Ann-Kristin Schmälter, Maud Ngoya, Jacques-Emmanuel Galimard, Ali Bazarbachi, Jürgen Finke, Nicolaus Kröger, Martin Bornhäuser, Matthias Stelljes, Friedrich Stölzel, Johanna Tischer, Thomas Schroeder, Peter Dreger, Igor-Wolfgang Blau, Bipin Savani, Sebastian Giebel, Jordi Esteve, Arnon Nagler, Christoph Schmid, Fabio Ciceri, Mohamad Mohty

**Affiliations:** 1 Department of Hematology and Oncology, Augsburg University Hospital and Medical Faculty, Bavarian Cancer Research Center (BZKF) and Comprehensive Cancer Center Augsburg, Augsburg, Germany; 2 EBMT Paris Study Unit, Department of Hematology and Cell Therapy, Hôpital Saint-Antoine, Paris, France; 3 Bone Marrow Transplantation Program, Department of Internal Medicine, American University of Beirut, Medical Center, Beirut, Libanon; 4 University of Freiburg, Department of Medicine, Hematology, Oncology, Freiburg, Germany; 5 University Medical Center Hamburg-Eppendorf, Department of Stem Cell Transplantation, Hamburg, Germany; 6 University Hospital Dresden, TU Dresden, Medizinische Klinik und Poliklinik I, Dresden, Germany; 7 University of Muenster, Department of Hematology and Oncology, Muenster, Germany; 8 University Hospital Schleswig-Holstein, Kiel, Department of Stem Cell Transplantation and Cellular Immunotherapies, Kiel University, Kiel, Germany; 9 University Hospital of Munich, Campus Grosshadern, Department of Internal Medicine III, Munich, Germany; 10 University Hospital Essen, Department of Hematology and Stem Cell Transplantation, Essen, Germany; 11 University of Heidelberg, Medizinische Klinik und Poliklinik V, Heidelberg, Germany; 12 Medizinische Klinik Hämatologie, Onkologie und Tumorimmunologie, Charité Universitätsmedizin Berlin, Berlin, Germany; 13 Department of Hematology and Oncology, Vanderbilt University Medical Center, Nashville, Tenn USA; 14 Department of Bone Marrow Transplantation and Hematology-Oncology, Maria Sklodowska-Curie Cancer Center and Institute of Oncology, Gliwice, Poland; 15 Hematology Department, Hospital Clinic Barcelona, Barcelona, Spain; 16 Hematology and Bone Marrow Transplantation Division, Chaim Sheba Medical Center, Tel Aviv University, Ramat Gan, Israel; 17 Unit of Hematology and BMT, IRCCS Ospedale San Raffaele, University Vita-Salute San Raffaele, Milano, Italy

**Keywords:** Stem-cell research, Acute myeloid leukaemia, Cancer stem cells

## Abstract

Second allogeneic stem cell transplantation (alloSCT2) is among the most effective treatments for acute myeloid leukemia (AML) relapse after first alloSCT (alloSCT1). Long-term EBMT registry data were used to provide large scale, up-to-date outcome results and to identify factors for improved outcome. Among 1540 recipients of alloSCT2, increasing age, better disease control and performance status before alloSCT2, more use of alternative donors and higher conditioning intensity represented important trends over time. Between the first (2000–2004) and last (2015–2019) period, two-year overall and leukemia-free survival (OS/LFS) increased considerably (OS: 22.5–35%, LFS: 14.5–24.5%). Cumulative relapse incidence (RI) decreased from 64% to 50.7%, whereas graft-versus-host disease and non-relapse mortality (NRM) remained unchanged. In multivariable analysis, later period of alloSCT2 was associated with improved OS/LFS (HR = 0.47/0.53) and reduced RI (HR = 0.44). Beyond, remission duration, disease stage and patient performance score were factors for OS, LFS, RI, and NRM. Myeloablative conditioning for alloSCT2 decreased RI without increasing NRM, leading to improved OS/LFS. Haploidentical or unrelated donors and older age were associated with higher NRM and inferior OS. In summary, outcome after alloSCT2 has continuously improved over the last two decades despite increasing patient age. The identified factors provide clues for the optimized implementation of alloSCT2.

## Introduction

Allogeneic stem cell transplantation (alloSCT) represents a potentially curative treatment for patients suffering from acute myeloid leukemia (AML). However, leukemia relapse after the first alloSCT (alloSCT1) occurs in 30–50% of patients [1, 2]. The prognosis of these patients is generally poor, especially if they relapse within six months from alloSCT1 [3, 4]. A second alloSCT (alloSCT2) has become one of the most frequently applied therapy in patients who are considered fit enough to tolerate the procedure [5]. In a recent study, the Acute Leukaemia Working Party (ALWP) of the European Society for Blood and Marrow Transplantation (EBMT) has analysed trends in treatment and outcome in more than 8000 patients experiencing AML relapse post alloSCT1 [6]. AlloSCT2, although performed only in a minority of patients, contributed considerably to long-term remission and improved outcome over time. However, a detailed analysis of alloSCT2 was not the focus of this study.

Therefore, we performed a detailed retrospective registry-based analysis among adults receiving alloSCT2 for AML relapse after alloSCT1 between the years 2000 and 2019. The study aimed to analyze trends in patient characteristics, transplant settings and outcome over the last two decades and to identify factors associated with improved results after alloSCT2, that might allow to define strategies for an optimized implementation of the procedure.

## Methods

### Study design

Data were extracted from the EBMT registry, which comprises >600 transplant centers providing reports and annual follow-up on all consecutive SCT. Audits are routinely performed to determine data accuracy. Since 1990, patients have provided informed consent, authorizing the use of their personal information for research purposes. The study was approved by the general assembly and review board of the ALWP and complied with country-specific regulatory requirements.

Considering all types of conditioning, all donors apart from cord blood, and all disease stages, 1540 consecutive patients ≥18 years receiving alloSCT2 for hematological AML relapse after alloSCT1 were included. AlloSCT2 applied for graft failure were excluded. For evaluation of changes over time, patients were grouped by period of transplant into 5-year intervals. Variables of interest included patient and donor characteristics (age, patient and donor sex, Karnofsky performance score (KPS), donor switch for alloSCT2, cytomegalovirus (CMV) serostatus of patient and donor), disease-related (de novo/secondary AML, cytogenetics, remission duration after alloSCT1, disease status at alloSCT2) and transplant-related factors (year of transplant, delay between relapse post alloSCT1 and alloSCT2, graft source, donor type, female donor to male recipient, conditioning regime, use of myeloablative or reduced intensity conditioning (MAC/RIC), total body irradiation (TBI), in-vivo or in-vitro T-cell depletion (TCD) or use of post-transplant cyclophosphamide (PTCY) and graft-versus-host disease (GVHD) prophylaxis). Analyzed outcome variables comprised overall survival (OS), leukemia-free survival (LFS), cumulative incidence of relapse (RI), non-relapse mortality (NRM), acute and chronic graft-versus-host disease (GVHD), and GVHD-free/relapse-free survival (GRFS).

### Definitions

As recommended [7], complete remission (CR) was defined by <5% bone marrow blasts, absence of circulating blasts and extramedullary disease. Failure to achieve CR after two courses of standard induction chemotherapy was defined as primary induction failure (PIF) [8]. Relapse was defined as more than 5% bone marrow blasts or reappearance of circulating blasts after a documented CR or development of extramedullary disease. OS was defined as the interval between day of alloSCT2 and day of death or last follow-up, LFS as interval between alloSCT2 and date of leukemia persistence, relapse, progression or death. NRM was defined as death from any cause without relapse or progression. GRFS was defined as survival without acute GVHD grades III–IV, chronic GVHD requiring systemic treatment, relapse, or death [9]. Reduced intensity conditioning (RIC) was defined using EBMT guidelines [8]. Cytogenetic subgroups were defined according to European Leukemia Net (ELN) criteria [10].

### Statistics

Descriptive statistics were presented using median, range (from minimum to maximum) and inter-quartile range for continuous data, frequency and percentages for categorical data. Survivors were censored at last contact. Cumulative incidence was used to estimate the endpoints of NRM, RI, acute and chronic GVHD to accommodate for competing risks. Relapse and death were considered competing events for acute and chronic GVHD. Probabilities of OS, LFS, and GRFS were calculated using the Kaplan–Meier method. The median follow-up has been estimated using the reverse Kaplan-Meier method. All outcomes have been censored at two years according to the median follow-up of the most recent period.

A Cox proportional-hazards model was performed for multivariable regressions. Period of transplant, type of alloSCT2, age, Karnofsky performance score (KPS) and disease stage at alloSCT2, cytogenetics, recipient/donor gender, cytomegalovirus (CMV) status, in-vivo T-cell depletion (TCD), conditioning, interval alloSCT1 to relapse, and interval relapse to alloSCT2 were included. Results were expressed as hazard ratio (HR) and 95% confidence intervals (CI). All tests were 2-sided. Center effect was considered as frailty. Type I error rate was fixed at 0.05 for factors associated with time-to-event outcomes. Analyses were performed using R 4.3.2 (https://www.R-project.org/).

## Results

### Trends in patient and transplant characteristics

Over time, the number of patients reported to have received an alloSCT2 in EBMT centers increased from 144 in the period 2000–2004 to 619 between 2015 and 2019, with remarkable changes with respect to patient characteristics and transplant settings: In recent time periods, patients were older (median age 43.4 years between 2000 and 2004 and 48.6 years between 2015 and 2019, *p* = 0.012), received alloSCT2 more frequently in CR (37.5% between 2000 and 2004, increase to 52.7% in 2015–2019, *p* < 0.0001) and had a better performance status before alloSCT2 (2000–2004: KPS ≥90% in 25.5% patients, 2015–2019: increase to 58.5%, p < 0.001). Moreover, the interval between alloSCT1 and relapse increased from 9.1 to 12.3 months (*p* < 0.0001) and the interval between relapse after alloSCT1 and alloSCT2 increased from 2.5 to 3.5 months (*p* < 0.001). In the more recent time periods, a different donor, especially unrelated (increase from 30.6% [2000–2004] to 61.7% [2015–2019]) and haploidentical (increase from 0.7% [2000–2004] to 22.9% [2015–2019]), was used more frequently for alloSCT2 (p < 0.0001 each). Finally, over time, a more frequent use of myeloablative conditioning (MAC; *p* = 0.045), in-vivo TCD (*p* < 0.001), as well as post-transplant cyclophosphamide (PTCY) (*p* < 0.001) and cyclosporin A/mycophenolate mofetil (MMF) for GVHD prophylaxis (*p* < 0.001) for alloSCT2 was observed. Cytogenetic subgroups were not equally distributed over the years, however without a clear trend. No differences were found regarding stem cell source, sex of patient and donor, frequency of female donors for male recipients, number of secondary AML, CMV status of donors, cell source, or use of total body irradiation (TBI) (see Table 1 and supplemental Table 1 for details).

**Table 1:** Changes in patient and second transplant characteristics over time.

		Total	2000–2004	2005–2009	2010–2014	2015–2019	*p*-value
		N = 1540	N = 144	N = 352	N = 425	N = 619	
Age at alloSCT2 (years)	Median (range) [IQR]	47.06 (18.6–74.8) [36.3–56.8]	43.4 (18.6–68.8) [33.6–56.1]	46.5 (18.9–72.7) [36.5–55.7]	46.9 (18.9–73.4) [36.6–56.3]	48.6 (19.2–74.8) [36.7–58]	0.012
Sex patient (%)	Female	728 (47.3)	68 (47.2)	166 (47.2)	203 (47.8)	291 (47)	0.996
	Male	812 (52.7)	76 (52.8)	186 (52.8)	222 (52.2)	328 (53)	
Karnofsky index (%)	< 90	591 (46.9)	35 (74.5)	150 (60)	163 (43.1)	243 (41.5)	< 0.001
	≥ 90	669 (53.1)	12 (25.5)	100 (40)	215 (56.9)	342 (58.5)	
	missing	280	97	102	47	34	
Secondary AML (%)	No	1346 (87.4)	134 (93.1)	303 (86.1)	373 (87.8)	536 (86.6)	0.161
	Yes	194 (12.6)	10 (6.9)	49 (13.9)	52 (12.2)	83 (13.4)	
Cyto-genetics (%)	good	73 (4.7)	10 (6.9)	20 (5.7)	17 (4)	26 (4.2)	< 0.001
	intermediate	650 (42.2)	64 (44.4)	154 (43.8)	155 (36.5)	277 (44.7)	
	poor	262 (17)	30 (20.8)	52 (14.8)	53 (12.5)	127 (20.5)	
	NA	555 (36)	40 (27.8)	126 (35.8)	200 (47.1)	189 (30.5)	
Interval between alloSCT1 and relapse (months)	median (range) [IQR]	10.22 (0.5–223.2) [4.7–23.6]	9.1 (1–117.3) [4.1–15.6]	8 (0.9–118.7) [4.3–18.9]	10.1 (0.5–223.2) [4.4–24.2]	12.3 (0.7–208.6) [5.3–27.9]	< 0.0001
Donor type at alloSCT1 (%)	MSD	751 (48.8)	108 (75)	208 (59.1)	209 (49.2)	209 (49.2)	Not done
	URD	741 (48.1)	36 (25)	140 (39.8)	205 (48.2)	205 (48.2)	
	Haplo	48 (3.1)	0 (0)	4 (1.1)	11 (2.6)	11 (2.6)	
Donor type at alloSCT2 (%)	MSD^a^	495 (32.1)	99 (68.8)	179 (50.9)	122 (28.7)	95 (15.3)	<0.001
	URD	818 (53.1)	44 (30.6)	155 (44)	237 (55.8)	382 (61.7)	
	Haplo^b^	227 (14.7)	1 (0.7)	18 (5.1)	66 (15.5)	142 (22.9)	
Same donor for alloSCT 1 and 2 (%)	No	670 (66.6)	22 (27.5)	72 (41.4)	109 (62.6)	467 (80.8)	< 0.001
	Yes	336 (33.4)	58 (72.5)	102 (58.6)	65 (37.4)	111 (19.2)	
	missing	534	64	178	251	41	
Female donor to male recipient at alloSCT2	No	1289 (84.4)	122 (85.3)	285 (81.9)	356 (84.2)	526 (85.8)	0.442
	Yes	238 (15.6)	21 (14.7)	63 (18.1)	67 (15.8)	87 (14.2)	
	missing	13	1	4	2	6	
Disease status at alloSCT2 (%)	CR	666 (43.2)	54 (37.5)	110 (31.2)	176 (41.4)	326 (52.7)	< 0.0001
	Relapse	874 (56.8)	90 (62.5)	242 (68.8)	249 (58.6)	293 (47.3)	
Molecular remission at alloSCT2 (%)	No	106 (38.1)	1 (16.7)	11 (42.3)	23 (34.3)	71 (39.7)	0.628
	Yes	172 (61.9)	5 (83.3)	15 (57.7)	44 (65.7)	108 (60.3)	
	missing	1262	138	326	358	440	
TBI (%)	No	1191 (78.1)	118 (83.7)	267 (76.5)	335 (80)	471 (76.6)	0.190
	Yes	333 (21.9)	23 (16.3)	82 (23.5)	84 (20)	144 (23.4)	
	Missing	16	3	3	6	4	
MAC (%)	No	839 (55.6)	83 (61.5)	202 (58.4)	239 (57.6)	315 (51.4)	0.045
	Yes	670 (44.4)	52 (38.5)	144 (41.6)	176 (42.4)	298 (48.6)	
	Missing	31	9	6	10	6	
In vivo T cell depletion (%)	No	707 (53.1)	51 (62.2)	154 (61.1)	223 (55.8)	279 (46.7)	<0.001
	Yes	624 (46.9)	31 (37.8)	98 (38.9)	177 (44.2)	318 (53.3)	
	Missing	209	62	100	25	22	
PTCY (%)	No	1112 (85.7)	76 (100)	239 (99.6)	343 (87.9)	454 (76.8)	<0.001
	Yes	185 (14.3)	0 (0)	1 (0.4)	47 (12.1)	137 (23.2)	
	Missing	243	68	112	35	28	

^a^no change of donor for alloSCT2 in nearly all patients.

^b^change of donor in nearly all patients.

*alloSCT2* second allogeneic stem cell transplantation, *IQR* interquartile range, *alloSCT1* first allogeneic stem cell transplantation, *AML* acute myeloid leukemia, *MSD* matched sibling donor, *URD* unrelated donor, *Haplo* haploidentical donor, *CR* complete remission, *TBI* total body irradiation, *MAC* myeloablative conditioning, *PTCY* post-transplant cyclophosphamide.

### Outcomes

**Fig. 1:**
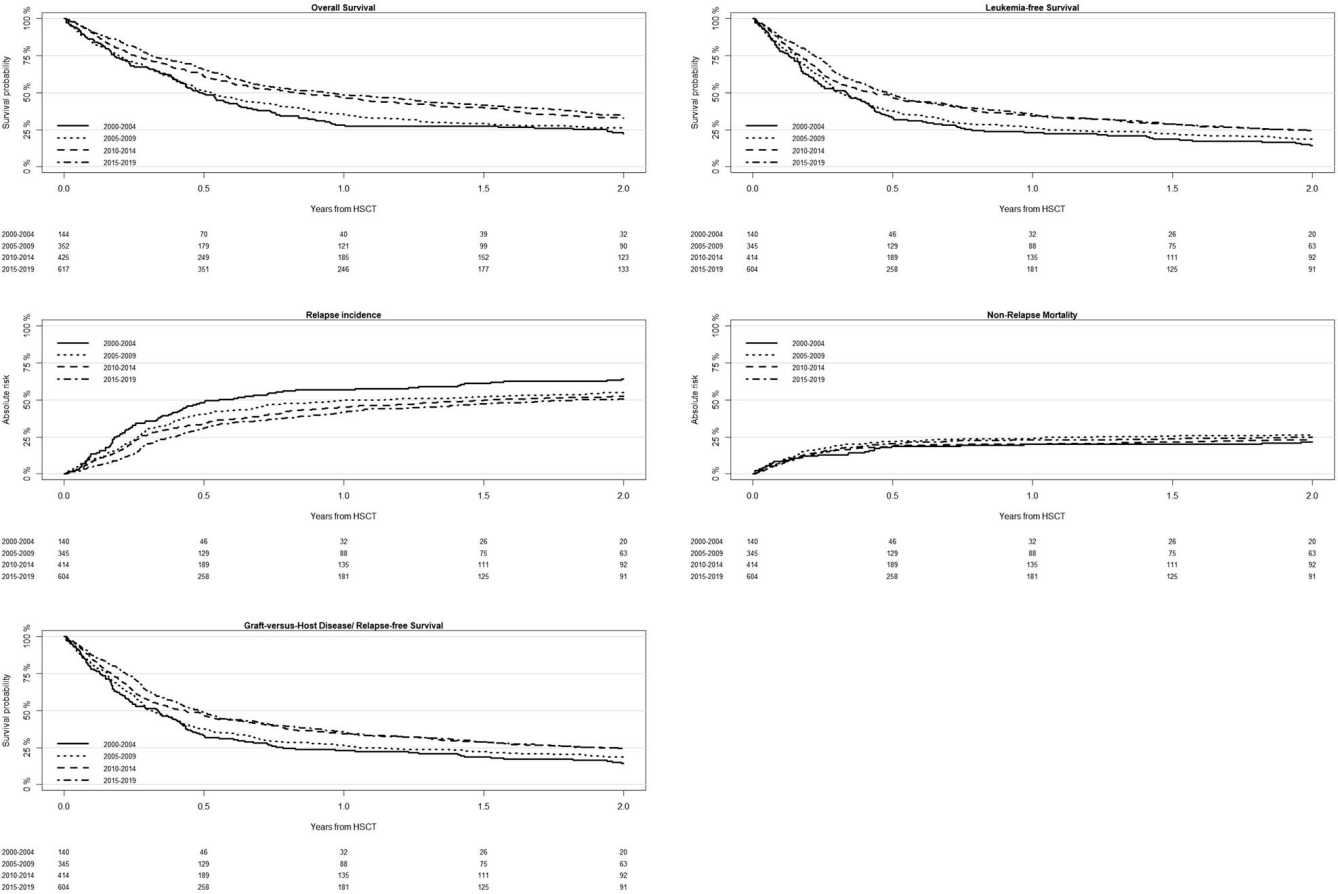
Outcomes over time after second allogeneic stem cell transplantation. Overall survival, leukemia-free survival, non-relapse mortality, the cumulative incidence of relapse and graft-versus-host disease/ relapse-free survival after second allogeneic stem cell transplantation for patients transplanted between 2000 and 2004, 2005 and 2009, 2010 and 2014, and 2015 and 2019.

Median follow-up from alloSCT2 was 15.1 years in the cohort transplanted between 2000 and 2004, and 2.5 years in the most recent cohort. Concerning clinical results after alloSCT2, RI decreased over time, whereas OS, LFS, and GRFS improved: Between 2000 and 2019, two-year OS increased from 22.5% to 35%, LFS increased from 14.5% to 24.5% and GRFS increased from 10.5% to 17%. Whereas no clear trend was observed for NRM and incidence of acute and chronic GVHD, RI decreased from 64% to 50.7% (Fig. 1, Table 2).

**Table 2:** Change in outcomes over time after second allogeneic stem cell transplantation.

Outcomes	Total	2000–2004	2005–2009	2010–2014	2015–2019
	Estimation (95 CI %)	Estimation (95 CI %)	Estimation (95 CI %)	Estimation (95 CI %)	Estimation (95 CI %)
Median FU (y)	4.9 (4.3–5.6)	15.1 (7.2–16.3)	11 (9.7–11.8)	6.9 (6.3–7.2)	2.5 (2.2–2.8)
OS (2 y)	31.2 (28.8–33.7)	22.5 (16–29.6)	26.4 (21.9–31.2)	32.9 (28.3–37.6)	35 (30.8–39.2)
LFS (2 y)	22.2 (20–24.4)	14.5 (9.2–20.9)	18.7 (14.7–23)	24.5 (20.3–28.8)	24.5 (20.8–28.4)
RI (2 y)	53.5 (50.9–56.1)	64 (55.3–71.4)	55.1 (49.7–60.2)	52.6 (47.6–57.4)	50.7 (46.3–54.9)
NRM (2 y)	24.3 (22.1–26.6)	21.5 (15.1–28.8)	26.2 (21.7–31)	22.9 (18.9–27.1)	24.8 (21.3–28.4)
aGVHD-II/IV (100 d)	31.7 (29.3–34.2)	30.2 (22.5–38.3)	36.1 (30.9–41.3)	32.2 (27.6–36.9)	29.1 (25.4–33)
aGVHD-III/IV (100 d)	15.5 (13.6–17.4)	13.2 (8–19.6)	16.1 (12.3–20.3)	17.6 (14–21.5)	14.2 (11.5–17.3)
GRFS (2 y)	15.5 (13.7–17.5)	10.5 (6.2–16.1)	12.6 (9.4–16.4)	17.5 (14–21.4)	17 (13.9–20.5)
cGVHD (2 y)	28.2 (25.9–30.7)	24.8 (17.7–32.5)	27.8 (22.9–32.8)	32.6 (28–37.4)	25.7 (22–29.5)
cGVHD Ext (2 y)	12.4 (10.7–14.2)	10.9 (6.2–17)	12.1 (8.8–16.1)	13.6 (10.3–17.3)	12 (9.3–15)

*FU* follow-up, *y* years, *OS* overall survival, *LFS* leukemia-free survival, *RI* Relapse incidence, *NRM* non-relapse mortality, *d* day, *a/c* acute/chronic, *GVHD* Graft-versus-Host Disease, *GRFS* Graft-versus-Host Disease/relapse-free survival, *Ext* extensive.

### Multivariable analysis of risk factors

In the multivariable analysis (Table 3 and supplemental Table 2), a later period of alloSCT2 was significantly associated with improved OS (2015–2019: HR 0.47, *p* < 0.001; 2010–2014: HR 0.48, *p* = 0.002; 2005–2009: HR 0.61, *p* = 0.04), LFS (2015–2019: HR 0.53, *p* = 0.01; 2010–2014: HR 0.5, *p* = 0.003; 2005–2009: HR 0.62, *p* = 0.046) and GRFS (2015–2019: HR 0.52, *p* = 0.003; 2010–2014: HR 0.47, *p* < 0.001; 2005–2009: HR 0.58, *p* = 0.02). These findings came along with a markedly lower RI (HR 0.44, *p* = 0.003 for the period 2015–2019), whereas NRM and acute GVHD remained unchanged. Disease status at alloSCT2 had significant effects on OS, LFS, RI and NRM (HR for active disease at alloSCT2 1.59, *p* < 0.001 for OS; HR 1.63, *p* < 0.001 for LFS; HR 1.91, *p* < 0.001 for RI; HR 1.35, *p* = 0.04 for NRM). Similarly, longer duration of CR after alloSCT1 was associated with better OS and LFS, lower RI and lower rates of NRM and acute GVHD (HR for lower remission duration 1.83, *p* < 0.001 for OS; HR 1.72, *p* < 0.001 for LFS; HR 1.86, *p* < 0.001 for RI; HR 1.46, *p* = 0.01 for NRM; HR 1.32, *p* = 0.02 for acute GVHD). In contrast, alloSCT2 from haploidentical or unrelated donors, and older patient age were associated with higher NRM (HR 1.88, *p* = 0.01 for haploidentical donor; HR 2.14, *p* < 0.001 for unrelated donor; HR 1.1, *p* < 0.001 for increasing age by 5-year intervals) and therefore inferior OS (HR 1.34, *p* = 0.02 for haploidentical donor; HR 1.32, *p* = 0.02 for unrelated donor; HR 1.05, *p* = 0.002 for increasing age).

**Table 3:** Multivariable analysis of risk factors for outcome after second allogeneic stem cell transplantation.

Variable	Level	LFS		OS		RI		NRM	
		HR (95% CI)	*p* value	HR (95% CI)	*p* value	HR (95% CI)	*p* value	HR (95% CI)	*p* value
Period of transplant	2000–2004	1		1		1		1	
	2005–2009	0.62 (0.39–0.99)	**0.046**	0.61 (0.38–0.98)	**0.04**	0.52 (0.3–0.91)	**0.02**	0.95 (0.34–2.7)	0.93
	2010–2014	0.5 (0.32–0.79)	**0.003**	0.48 (0.3–0.76)	**0.002**	0.41 (0.24–0.72)	**0.002**	0.73 (0.26–2.05)	0.55
	2015–2019	0.53 (0.34–0.83)	**0.01**	0.47 (0.3–0.74)	**<0.001**	0.44 (0.25–0.76)	**0.003**	0.77 (0.27–2.15)	0.61
Type of donor for alloSCT2	MSD	1		1		1		1	
	Haplo	1.13 (0.89–1.44)	0.30	1.34 (1.04–1.73)	**0.02**	0.93 (0.68–1.28)	0.66	1.88 (1.2–2.97)	**0.01**
	URD	1.13 (0.92–1.38)	0.26	1.32 (1.05–1.66)	**0.02**	0.89 (0.69–1.14)	0.35	2.14 (1.42–3.23)	**<0.001**
Age at alloSCT2 (by 5 years)		1.02 (1–1.05)	0.11	1.05 (1.02–1.08)	**0.002**	1 (0.96–1.03)	0.86	1.1 (1.04–1.16)	**<0.001**
Disease status at alloSCT2	CR	1		1		1		1	
	Rel	1.63 (1.39–1.91)	**<0.001**	1.59 (1.34–1.88)	**<0.001**	1.91 (1.56–2.34)	**<0.001**	1.35 (1.02–1.8)	**0.04**
Cyto-genetics	Good	1		1		1		1	
	Interm	1.04 (0.71–1.53)	0, 85	1.09 (0.72–1.67)	0,68	1.14 (0.7–1.86)	0, 59	0.85 (0.43–1.71)	0, 65
	Poor	1.26 (0.84–1.89)	0, 26	1.4 (0.9–2.18)	0, 14	1.39 (0.84–2.31)	0, 20	1.06 (0.51–2.2)	0, 87
	NA	1.12 (0.76–1.66)	0, 56	1.18 (0.77–1.81)	0, 45	1.13 (0.69–1.85)	0, 63	1.08 (0.54–2.17)	0, 82
Female donor to male recipient	No	1		1		1		1	
	Yes	1.06 (0.87–1.28)	0, 58	0.94 (0.76–1.16)	0, 55	0.99 (0.78–1.26)	0, 92	1.13 (0.79–1.6)	0, 51
CMV status donor	Negative	1		1		1		1	
	Positive	1.04 (0.89–1.2)	0, 64	1.09 (0.93–1.28)	0, 26	1.02 (0.84–1.23)	0, 87	1.05 (0.81–1.37)	0, 71
In vivo T-cell depletion	No	1		1		1		1	
	Yes	0.92 (0.78–1.1)	0.37	0.89 (0.74–1.07)	0.20	1.05 (0.84–1.3)	0.68	0.7 (0.51–0.95)	**0.02**
Myelo-ablative regimen	No	1		1		1		1	
	Yes	0.79 (0.68–0.92)	**0.002**	0.82 (0.7–0.97)	**0.02**	0.7 (0.58–0.85)	**<0.001**	1.1 (0.84–1.44)	0.47
TBI	No	1		1		1		1	
	Yes	0.99 (0.83–1.18)	0, 89	1.05 (0.87–1.26)	0, 64	1 (0.79–1.26)	0, 99	0.95 (0.7–1.3)	0, 76
Karnofsky Index	< 90	1		1		1		1	
	≥ 90	0.84 (0.72–0.97)	**0.02**	0.74 (0.63–0.87)	**<0.001**	0.95 (0.78–1.15)	0.58	0.65 (0.49–0.85)	**0.002**
Interval alloSCT1 to 1st relapse	≥10.2mo	1		1		1		1	
	<10.2mo	1.72 (1.48–2)	**<0.001**	1.83 (1.56–2.15)	**<0.001**	1.86 (1.54–2.24)	**<0.001**	1.46 (1.11–1.93)	**0.01**
Interval relapse to alloSCT2 in mo		1.01 (0.98–1.03)	0, 70	1 (0.97–1.03)	0, 96	1.01 (0.98–1.04)	0,54	0.99 (0.95–1.04)	0,76

*LFS* leukemia free survival, *OS* overall survival, *RI* relapse incidence, *NRM* non-relapse mortality, *HR* hazard ratio, *CI* confidence interval, *alloSCT* allogeneic stem cell transplantation, *alloSCT1* first SCT, *alloSCT2* second SCT, *CR* complete remission, *Rel* relapse, *mo* months, *CMV* cytomegalo virus, *TBI* total body irradiation, *MSD* matched sibling donor, *Haplo* haploidentical, *URD* unrelated donor, *interm* intermediate, *NA* not available.

Significant values are in bold.

MAC was associated with decreased RI without increasing NRM, leading to increased OS and LFS (HR 0.7, *p* < 0.001 for RI; HR 1.1, *p* = 0.47 for NRM; HR 0.82, *p* = 0.02 for OS; HR 0.79, *p* = 0.002 for LFS). LFS and OS were positively affected by a better KPS (HR 0.84, *p* = 0.02 for LFS; HR 0.74, *p* < 0.001 for OS). In-vivo TCD was associated with lower NRM and lower rates of GVHD (HR 0.7, *p* = 0.02 for NRM; HR 0.52, *p* < 0.001 for acute GVHD grades II–IV; HR 0.64, *p* = 0.003 for chronic GVHD).

### Role of donor switch for alloSCT2

Switch to another donor could not be included in the main multivariable model due to missing information in about one third of patients. However, an exploratory analysis of 1006 informative patients revealed no signal of a significant influence (data not shown).

## Discussion

This large registry analysis describes trends in patient characteristics, transplant strategies, and outcome after alloSCT2 for patients with AML relapsing after a first alloSCT over the last two decades. As shown for alloSCT1 [11], the number of second transplants has increased considerably over time, which may be due to an easier availability of alternative donors such as matched unrelated (MUD) and haploidentical donors. The formation of different RIC regimen [12] and improved supportive care might be other reasons [13]. Further significant changes over time comprised increasing patient age, longer remission after alloSCT1, more frequent use of alternative donors and donor change for alloSCT2, changes in GVHD prophylaxis, more intensive conditioning as well as improved disease control and improved KPS at alloSCT2. Fortunately, 2-year OS after alloSCT2 has continuously increased over time, reaching 35% in the most recent cohort. This was mainly due to a marked decrease in 2-year RI. In contrast, both rates of 2-year NRM and GVHD remained stable over the years, despite increased patient age and more frequent use of alternative donors. Better performance score at alloSCT2 might have counterbalanced the increased risk for NRM associated with increasing age and alternative donors. Besides alloSCT2 in earlier years, identified risk factors for inferior outcome after alloSCT2 included established variables such as older age, shorter remission after alloSCT1, donor type other than a matched sibling donor (MSD), RIC for alloSCT2, as well as active disease and lower KPS at time of alloSCT2.

Considering the analysis of changes over time together with risk factors for outcome, some lessons can be learned for planning and performing alloSCT2 for AML relapse after alloSCT1. These strategies include both measures to be taken before, during, and after alloSCT2:

The duration of remission between alloSCT1 and relapse is probably the most relevant risk factor for outcome, as seen in our analysis as well as in previous studies [5, 6, 14–16]. Early relapse is thought to mainly represent an aggressive nature of the leukemia with low sensitivity to both the conditioning and the allogeneic immune reaction [17]. However, due to the limited time for recovery from the physical and mental toxicities of alloSCT1, the need for salvage therapy shortly after alloSCT1 might also increase toxicity, decrease patients’ general conditions and hence diminish the resilience to another transplant, as well as their motivation to undergo the procedure for a second time. Therefore, prolongation of the remission after alloSCT1 is of benefit also for those finally developing relapse. More frequent use of maintenance therapy in recent years might be an explanation for the prolonged remission observed in the later period of our study, although we cannot prove this from our data, since the treatment applied between alloSCT1 and 2 is not covered by the EBMT registry. Nevertheless, both targeted therapies [18–20], unspecific pharmaceutical approaches [21–24] and prophylactic or preemptive donor lymphocyte infusion [25–27] have been increasingly used for maintenance post-transplant after showing their potential to reduce the relapse rate and lengthen remission duration after alloSCT.

Second, our data as well as observations from other studies [14, 17, 28] including a recent meta-analysis [16], showed that initial disease control and, at best, achieving CR after post-transplant relapse is a major factor for final success of alloSCT2. Therefore, timely and effective medical treatment shortly after diagnosis of post-transplant relapse is mandatory, which, however, must avoid disproportionate side effects, to which patients in this situation are highly sensitive. Carefully adapting treatment toxicity to the individual patient is of particular relevance, given that a better KPS was another factor independently associated with outcome from alloSCT2 in the present study. As a limitation of our analysis, we did not have enough details on therapies applied between relapse and alloSCT2 in the registry to draw meaningful conclusions concerning the optimized strategy. Fortunately, modern antileukemic therapies offer a better balance between efficacy and toxicity than classical chemotherapy. In particular, the use of hypomethylating agents±venetoclax [29–31] or application of targeted therapies [32–37] represent promising options in that sense.

Third, since despite recent achievements, long-term remission is a rarity after conventional treatment for post-transplant relapse, the identification of a donor for an eventual alloSCT2 should be part of the management immediately after relapse, to allow alloSCT2 at the optimal time point, defined by best disease response and a high KPS. Although intuitive, change to an alternative donor for alloSCT2 does not seem to be mandatory, since a variety of studies including our own could not demonstrate an advantage (although no disadvantage either) after alloSCT2 from a different donor [14–16]. Due to missing information in one third of patients, donor switch could not be included in the multivariate analysis in our study. However, an exploratory analysis of 1006 patients revealed no hint of a significant influence, suggesting that the observed trend to more frequent donor change in recent years did not decisively contribute to improved outcome over time. In contrast, an improved outcome was observed among patients receiving alloSCT2 from a MSD, who in the vast majority did not undergo donor change. This indirectly might suggest that at least after alloSCT1 from a MSD, using the same donor for alloSCT2 remains an option. Nevertheless, beyond unavailability of the original donor, switching to a different one might be reasonable under certain conditions. For instance, the situation of HLA loss by the malignant blasts, particularly after haploidentical or mismatched unrelated alloSCT1 [38], justifies change to an alternative donor [39], although unequivocal clinical evidence is missing. Furthermore, as described above, optimized timing for alloSCT2 to the point of maximum control of the leukemia and the patient being in good clinical condition, is mandatory. In that sense, the most easily available donor, including mismatched relatives, might be preferable, given that the general feasibility of alloSCT2 from a haploidentical donor has been demonstrated [40, 41].

Forth, according to our data, a MAC regimen should be considered for alloSCT2 in all patients who might tolerate it. Similar findings have been described by others [28] and are supported by data obtained both after alloSCT1 [42] and in a prospective trial in alloSCT2 [43]. Recent developments in the field of conditioning have identified myeloablative regimens with reduced toxicity and hence improved outcome [44]. Beyond, although not validated for alloSCT2, the EBMT transplant conditioning intensity (TCI) score [12] may support the selection of less toxic, but still myeloablative regimen also for alloSCT2. Unfortunately, the huge variety of conditioning regimens applied in our cohort precluded a reasonable comparison and hence the definition of an optimized regimen.

Finally, an optimized GVHD prophylaxis might improve the results after alloSCT2. In our analysis, the use of in-vivo TCD was associated with a markedly decreased incidence of both acute and chronic GVHD, leading to a reduction of NRM. Although this did not translate into improved survival, using in-vivo TCD might be preferable in alloSCT2, rather than omitting it in the interest of an eventually stronger graft-versus-leukemia effect. The use of PTCY might be an alternative to in-vivo TCD, even in the matched donor setting. However, since this strategy has only been introduced very recently, data are not mature enough to draw any conclusion on its use in the setting of alloSCT2.

Apart from the retrospective nature, several limitations of our study need to be considered. As discussed above, we lack sufficient data on maintenance therapy after alloSCT1 and initial disease control strategies after post-transplant relapse. Hence, the higher percentage of patients receiving alloSCT2 in CR and good clinical status in the later periods might be a consequence of the more frequent use of modern targeted therapies. Beyond, we missed information on the quality of remission before alloSCT2, since minimal residual disease (MRD) status was reported only recently and therefore in about 20% of our patients. Data from alloSCT1 have shown an advantage for patients transplanted in MRD-negative CR [45, 46], although this seems not to be true for all molecular markers used for MRD detection [47]. Hence the role of MRD negativity before alloSCT2 remains to be elucidated. As discussed above, the influence of PTCY as GVHD prophylaxis could not be evaluated either in the multivariable analysis because it was mainly used in the later period. Last, the contribution of maintenance treatment after alloSCT2 to overall outcome cannot be estimated since these data have not been captured in the registry for many years.

In summary, according to this large registry analysis on >1500 patients, outcome after alloSCT2 has continuously improved over the last two decades, despite increasing patient age. In particular, decreased RI did not come at the cost of increased toxicity. This might be a result of better disease control and improved performance score at time of alloSCT2, as well as an increasing use of MAC, in-vivo TCD and eventually PTCY in alloSCT2 over time. These data encourage to perform alloSCT2 in relapsed AML after first transplant. Factors that were associated with improved outcome may help to optimize the procedure, which at present represents the most effective therapy in this setting. In detail, maintenance strategies after alloSCT1, early identification of a donor for alloSCT2, application of less toxic strategies for initial disease control, timely implementation of alloSCT2 when the best response status has been achieved, as well as the use of intensive, but toxicity-reduced conditioning regimen and in-vivo T-cell depletion for GVHD prophylaxis are treatment elements that might contribute to improved results.

Nevertheless, with still half of the patients relapsing again and only one third being cured by alloSCT2, there is still a lot of room for improvement. Preclinical research has largely increased our understanding of the biology of post-transplant relapse [48, 49]. It is hoped that specific and individualized treatment will be based on this knowledge in the future to improve outcomes after relapse after alloSCT.

### Supplementary information

Supplement

## Data Availability

The data were allocated by the EBMT registry in Paris. The datasets are available upon data-specific request.
