# Supplementary material for: Continuously improving outcome over time after second allogeneic stem cell transplantation in relapsed acute myeloid leukemia: an EBMT registry analysis of 1540 patients
Source: Blood Cancer J. 2024 May 2;14(1):76. doi: 10.1038/s41408-024-01060-4 (PMC11066014; doi:10.1038/s41408-024-01060-4)
Supplement: Supplementary file 1 — Supplement [file 41408_2024_1060_MOESM1_ESM.pdf]

Supplement

Supplemental Table 1. Additional details on changes in patient and second transplant characteristics over time

		Total	2000-2004	2005-2009	2010-2014	2015-2019	p-value
		N=1540	N=144	N=352	N=425	N=619	
**Year of alloSCT2**	median [IQR]	2013 [2008-2016]	2003 [2001-2004]	2007 [2006-2008]	2012 [2011-2013]	2017 [2016-2018]	
**Interval between relapse post alloSCT1 and alloSCT2 (months)**	median (range) [IQR]	2.99 (0-17.8) [1.8-4.8]	2.5 (0.2-15.1) [1.2-3.9]	2.2 (0.3-15.5) [1.2-3.6]	3.2 (0.2-16.6) [1.9-5.2]	3.5 (0-17.8) [2.3-5.4]	< 0.001
**Sex donor at alloSCT2 (%)**	Female	523 (34.6)	45 (32.1)	133 (38.7)	132 (31.6)	213 (34.9)	0.205
	Male	990 (65.4)	95 (67.9)	211 (61.3)	286 (68.4)	398 (65.1)	
	missing	27	4	8	7	8	
**CMV status patient (%)**	Negative	484 (34.6)	35 (38.5)	115 (40.5)	151 (36.5)	183 (30.1)	0.012
	Positive	913 (65.4)	56 (61.5)	169 (59.5)	263 (63.5)	425 (69.9)	
	missing	143	53	68	11	11	
**CMV status donor (%)**	Negative	667 (48.2)	43 (49.4)	123 (43.8)	214 (52.2)	287 (47.4)	0.168
	Positive	717 (51.8)	44 (50.6)	158 (56.2)	196 (47.8)	319 (52.6)	
	missing	156	57	71	15	13	
**Cell source for alloSCT2 (%)**	BM	113 (7.3)	8 (5.6)	23 (6.5)	38 (8.9)	44 (7.1)	0.447
	PB	1427 (92.7)	136 (94.4)	329 (93.5)	387 (91.1)	575 (92.9)	
**In vitro T cell depletion (%)**	No	1456 (97.1)	129 (97)	324 (95.6)	398 (95.9)	605 (98.7)	0.009
	Yes	44 (2.9)	4 (3)	15 (4.4)	17 (4.1)	8 (1.3)	
	missing	40	11	13	10	6	
**Conditioning regimen (%)**	BuCy based	78 (5.4)	4 (3.5)	22 (7.2)	23 (5.6)	29 (4.7)	Not done
	BuFlu based	222 (15.3)	12 (10.6)	14 (4.6)	66 (15.9)	130 (21.1)	
	Flamsa based	83 (5.7)	0 (0)	11 (3.6)	34 (8.2)	38 (6.2)	
	FluMel based	192 (13.3)	9 (8)	40 (13.1)	58 (14)	85 (13.8)	
	TBI based	337 (23.3)	60 (53.1)	113 (37)	97 (23.4)	67 (10.9)	
	Treo based	333 (23)	23 (20.4)	82 (26.9)	84 (20.3)	144 (23.4)	
	Other combinations	202 (14)	5 (4.4)	23 (7.5)	52 (12.6)	122 (19.8)	
	missing	93	31	47	11	4	
**GVHD prophylaxis (%)**	CSA + MMF based	484 (36.4)	8 (9.8)	57 (22.6)	150 (37.5)	269 (45.1)	<0.001
	CSA +MTX based	261 (19.6)	26 (31.7)	60 (23.8)	78 (19.5)	97 (16.3)	
	CSA based	249 (18.7)	39 (47.6)	82 (32.5)	69 (17.2)	59 (9.9)	
	MMF based	202 (15.2)	0 (0)	20 (7.9)	63 (15.8)	119 (20)	
	other	134 (10.1)	9 (11)	33 (13.1)	40 (10)	52 (8.7)	
	missing	210	62	100	25	23	

Abbreviations: alloSCT2: second allogeneic stem cell transplantation, IQR: interquartile range, alloSCT1: first allogeneic stem cell transplantation, CMV: cytomegalovirus, PB: peripheral blood, BM: bone marrow, Bu: busulfan, Cy: cyclophosphamide, Flu: fludarabine, Mel: melphalan, Treo: treosulfan, GVHD: Graft-versus-host disease, CSA: ciclosporin A, MMF: mycophenolate mofetil, MTX: methotrexate

Supplemental Table 2. Multivariable analysis of risk factors for GRFS and GVHD after second allogeneic stem cell transplantation

Variable	Level	GRFS		Acute GVHD °II-IV		Chronic GVHD	
		HR (95% CI)	p value	HR (95% CI)	p value	HR (95% CI)	p value
**Period of transplant**	2000-2004	1		1		1	
	2005-2009	0.58 (0.38-0.9)	**0.02**	1.09 (0.51-2.34)	0,82	0.5 (0.22-1.15)	0,10
	2010-2014	0.47 (0.31-0.72)	**<0.001**	1.01 (0.48-2.13)	0,98	0.43 (0.19-0.97)	**0,04**
	2015-2019	0.52 (0.34-0.8)	**0.003**	0.87 (0.41-1.85)	0,73	0.28 (0.12-0.64)	**0,002**
**Type of donor for alloSCT2**	MSD	1		1		1	
	Haplo	1.01 (0.81-1.26)	0.95	0.83 (0.55-1.25)	0,38	0.8 (0.51-1.24)	0,32
	URD	1.21 (1-1.47)	0.06	2.14 (1.54-2.96)	**<0.001**	1.17 (0.83-1.64)	0,37
**Age at alloSCT2 (by 5 years)**		0.98 (0.96-1.01)	0.20	0.89 (0.85-0.93)	**<0.001**	0.97 (0.93-1.02)	0,25
**Disease status at alloSCT2**	CR	1		1		1	
	Rel	1.62 (1.39-1.87)	**<0.001**	1.42 (1.11-1.82)	**0,01**	1.17 (0.89-1.54)	0,27
**Cyto-genetics**	good	1		1		1	
	interm	1.26 (0.86-1.83)	0,23	1.38 (0.76-2.5)	0,29	0.93 (0.5-1.73)	0,82
	poor	1.66 (1.12-2.46)	**0,01**	1.41 (0.75-2.64)	0,29	1 (0.51-1.95)	1,00
	NA	1.37 (0.94-2.01)	0,10	1.17 (0.64-2.16)	0,61	0.98 (0.52-1.84)	0,95
**Female donor to male recipient**	No	1		1		1	
	Yes	1.19 (0.99-1.42)	0,06	1.16 (0.86-1.57)	0,33	1.2 (0.86-1.68)	0,28
**CMV status donor**	negative	1		1		1	
	positive	1.06 (0.92-1.22)	0,40	1.03 (0.81-1.3)	0,82	0.98 (0.76-1.27)	0,88
**In vivo T-cell depletion**	No	1		1		1	
	Yes	0.79 (0.67-0.93)	**0.004**	0.52 (0.4-0.68)	**<0.001**	0.64 (0.48-0.86)	**0,003**
**Myelo-ablative regimen**	No	1		1		1	
	Yes	0.85 (0.74-0.98)	**0.02**	0.91 (0.72-1.15)	0,41	1.13 (0.87-1.47)	0,37
**TBI**	No	1		1		1	
	Yes	1.06 (0.9-1.25)	0,48	0.99 (0.76-1.3)	0,97	0.95 (0.7-1.29)	0,74
**Karnofsky Index**	< 90	1		1		1	
	≥ 90	0.88 (0.76-1.01)	0.07	1.01 (0.8-1.29)	0,91	1.01 (0.78-1.32)	0,94
**Interval alloSCT1 to 1st relapse**	≥10.2mo	1		1		1	
	<10.2mo	1.58 (1.37-1.82)	**<0.001**	1.32 (1.04-1.68)	**0,02**	1.12 (0.86-1.46)	0,40
**Interval relapse to alloSCT2 in mo**		0.99 (0.96-1.01)	0,34	0.96 (0.92-1)	0,08	0.98 (0.94-1.03)	0,44

Abbreviations: GRFS: graft-versus-host disease/relapse free survival, GVHD: graft-versus-host disease, HR: hazard ratio, CI: confidence interval, alloSCT: allogeneic stem cell transplantation, alloSCT1: first SCT, alloSCT2: second SCT, CR: complete remission, Rel: relapse, mo: months, CMV: cytomegalo virus, TBI: total body irradiation, MSD: matched sibling donor, Haplo: haploidentical, URD: unrelated donor, interm: intermediate, NA: not available
